# Curbing inappropriate *C. difficile* treatment in patients receiving concomitant laxatives

**DOI:** 10.1017/ash.2025.25

**Published:** 2025-02-17

**Authors:** Paige Fields, Christopher David, Anish Choksi, Natasha N. Pettit, Alison K. Lew, Jennifer Pisano, Cynthia T. Nguyen

**Affiliations:** 1 Department of Pharmacy, University of Chicago Medicine, Chicago, IL, USA; 2 Department of Medicine, Section of Infectious Diseases and Global Health, University of Chicago, Chicago, IL, USA

## Abstract

In the setting of universal *Clostridioides difficile* screening, we implemented an alert that triggered when *C. difficile* treatment was ordered in patients who recently received laxatives. This resulted in *C. difficile* treatment avoidance in 37% of patients and was associated with drug cost savings of $143,905 over a 10-month period.

## Background

*Clostridioides difficile* (*C. difficile*) infection (CDI) is a heterogeneous disease ranging from asymptomatic carrier status to life-threatening colitis. Many providers order CDI treatment in response to diarrhea. However, hospital-associated diarrhea has many potential infectious and noninfectious causes, with ≤20% of cases attributable to *C. difficile.* ^1^ One of these iatrogenic causes is laxative use. Previous studies indicate that 19–44% of hospital inpatients tested for *C. difficile* received a laxative in the 48 hours prior.^2,3^

The nucleic acid amplification test (NAAT) detects *C. difficile* regardless of active infection.^4,5^ To increase the clinical relevance of a positive NAAT, national guidelines recommend ordering the NAAT only on patients experiencing at least three unformed stools in 24 hours, with symptoms not attributable to other causes.^5^ Unfortunately, this diagnostic algorithm is circumvented at hospitals (like ours) that universally screen patients for *C. difficile* colonization upon admission.

In June 2023, we implemented an Electronic Health Record (EHR) alert that triggered when oral vancomycin or fidaxomicin was ordered for a patient who received laxatives in the prior 48 hours. The alert recommended to remove the order for *C. difficile* treatment, discontinue the laxative(s), and monitor the patient’s stool output for an additional 48 hours. The objective of this study was to evaluate the impact of this alert on initiation of CDI treatment, in the setting of universal *C. difficile* screening.

## Methods

We performed a single-center, quasi-experimental, retrospective cohort study evaluating adult patients with an order for either oral (PO) vancomycin or fidaxomicin, and received a laxative within 48 hours prior to the CDI treatment order. A 10-month pre-intervention control period (3/1/22 – 12/31/22) was compared to a 10-month post-intervention period (6/1/23 – 3/31/24).

Aside from the alert, there were no changes to CDI diagnostic testing or treatment recommendations during the study period. Throughout the study period, the hospital protocol was to screen all patients for *C. difficile* colonization upon admission using a *C.difficile* NAAT on a rectal swab. Patients who tested positive were placed on contact precautions. Testing for suspected infection was performed on stool samples using the NAAT, using either a *C. difficile* assay alone or a gastrointestinal (GI) pathogen panel. If the patient received laxatives or had fewer than three liquid stools in the past 24 to 48 hours, testing was not recommended. This guidance was described within the CDI NAAT order. The order did not include patient-specific data on the receipt of laxatives. Pre-authorization was required to order the CDI NAAT test in patients hospitalized over 48 hours. If there was a positive admission rectal swab and the patient later developed symptoms consistent with CDI, providers were advised to treat CDI without repeat testing. No restrictions for ordering oral vancomycin or fidaxomicin were in place throughout the study period. Additionally, the Antimicrobial Stewardship Program (ASP) prospectively reviewed and audited all of patients started on CDI treatment.

In the post-intervention group, the EHR alert triggered when oral vancomycin or fidaxomicin was ordered in a patient who had a documented administration of a laxative within the prior 48 hours. The alert stated “This patient has received laxatives within the last 48 hours. Please discontinue any active laxative orders. It is recommended to NOT treat *C. difficile* until stool frequency is reassessed after 48 hours off of laxatives. If the patient has three or more unformed and unexplained stool within 24 hours, you may treat for *C. difficile*-associated diarrhea.” Within the alert, the provider could remove the CDI treatment order and/or discontinue laxative orders, or bypass the alert without making any changes.

The alert was considered dismissed if the provider proceeded with CDI treatment initiation (with or without the discontinuation of laxatives). The alert was considered accepted if laxative orders were discontinued and the CDI treatment order was removed. Accepted alerts were classified into delayed or avoided CDI courses. Courses were considered delayed if CDI treatment was initiated ≥48 hours after original alert triggered and considered avoided if no CDI treatment was received during the index hospitalization.

## Results

A total of 280 patients were included, 153 patients in the pre-intervention group and 127 in the post-intervention group. Results are outlined in Tables 1 and 2. A total of 127 alerts activated in the post-intervention group. Half (52%) of alerts were dismissed. CDI treatment was avoided in 47 (37%) patients. Fourteen (11%) of patients had a delay in CDI treatment. None of these patients later required an ICU admission for *C. difficile*, colectomy, or escalation to fulminant *C. difficile* treatment.

**Table 1.** Demographics and *C. difficile* treatment information

	Pre-intervention N = 153	Post-intervention N = 127
Age, years	59.6 ± 17.6	59.3 ± 17.9
Male, n (%)	78 (46)	69 (54)
IBD, n (%)	6 (4)	3 (2)
Cancer, n (%)	42 (27)	24 (19)
*C. difficile* test^a^ timing, n (%)		
Within 24 hours after admission	87 (57)	67 (53)
24–48 hours after admission	35 (23)	28 (22)
>48 hours after admission	31 (20)	32 (25)
No test during admission	0 (0)	5 (4)
*C. difficile* test^a^ specimen type, n (%)		
Rectal swab	124 (81)	96 (76)
* C. difficile* assay	23 (15)	16 (13)
GI Panel	6 (4)	10 (8)
Total vancomycin (oral) doses	3042	1537
Total fidaxomicin doses	951	568
Total bezlotoxumab doses	16	2
Total days of treatment, median (IQR)	10 (6)	10 (6)
Inpatient, median (IQR)	4 (6)	6 (7)
Outpatient, median (IQR)	5 (7)	7 (5)
*C. difficile* treatment courses per 100 patients	100	63
Total *C. difficile* doses per 100 patients		
Fidaxomicin doses per 100 patients	621	447
Vancomycin (oral) doses per 100 patients	1988	1210
Mortality (90-day CDI-related), n (%)	1 (0.7)	0 (0)
Readmission (90-day CDI-related), n (%)	2 (1.3)	3 (2.4)

a Refers to the CDI test completed prior to or within 24 hours after treatment initiation.

**Table 2.** Alert responses

Total # of alert triggers	127
Responses	
Dismissed (provider proceeded without change), n (%)	66 (52)
CDI treatment delayed ≥48 hours after alert, n (%)	14 (11)
CDI treatment avoided, n (%)	47 (37)
Vancomycin (oral)	29/47 (62)
Fidaxomicin	18/47 (38)
Estimated cost savings over 10 months^a^	$143,905.60

a Based on AWP, assuming vancomycin 125 mg orally q6h × 10 days ($1252.40/course) or fidaxomicin 200 mg orally q12h × 10 days ($5977/course)

We observed a 39% decrease in oral vancomycin and 28% decrease in oral fidaxomicin doses received when standardized to 100 patients. Median duration of treatment did not differ. There was no differences in 90-day CDI-related mortality (0.7% vs 0%, *P* > .999) or readmission (1.3% vs 2.4%, *P* = .662).

Drug cost savings were estimated based on the Average Wholesale Price (AWP) for a 10-day course of either oral vancomycin ($1252.40/course) or fidaxomicin ($5977/course). CDI treatment was avoided in 47 total patients: 29 of those being oral vancomycin courses and 18 fidaxomicin courses (based on the medication ordered), resulted in a drug cost savings of $143,905.60 over the 10-month study period.

## Discussion

In the setting of universal *C. difficile* screening, an alert targeting *C. difficile* treatment in patients receiving concomitant laxatives was associated with CDI treatment avoidance and drug cost savings. To our knowledge, this is the first report of an alert targeting *C. difficile* treatment. Despite concerns regarding alert fatigue, we found this alert to be highly effective, as 48% of alerts resulted in either CDI treatment delay or avoidance. We opted for a drug-based alert due to our use of the *C. difficile* screening test upon admission. Prescribers often decide to start CDI treatment based on the screening test result, rather than sending an additional test. In addition to drug cost savings, treatment avoidance has downstream effects, leading to preservation of resources. For example, since CDI treatment was not ordered, the ASP was not alerted to review the patient for prospective audit with feedback. Additionally, medication preparation, delivery, and administration time is preserved.

Our study has inherent limitations. First, our CDI diagnostic testing and treatment algorithm may differ from other centers. Our use of the NAAT test and screening patients upon admission may lead to over diagnosis and overtreatment, which may have enhanced the impact of our intervention.^4^ Second, we are unable to objectively describe why prescribers dismissed the alert. Anecdotally, prescribers often had a high clinical suspicion for CDI and/or the diarrhea started prior to laxative administration. Third, we did not collect the number of stools to confirm the diagnosis of each case. Finally, we did not delineate between inpatient and outpatient drug cost savings. Some patients do not complete CDI treatment while inpatient, so the cost savings directly observed by the hospital is likely less than the estimate.

In conclusion, implementation of an alert upon order entry of CDI treatment in patients who received laxative(s) within the previous 48 hours, resulted in CDI treatment avoidance in 37% of patients and an estimated cost savings of $143,905.60. Given the heterogeneity of CDI diagnostic testing and management, further evaluation is necessary to determine the impact of such an intervention at other centers.
